# GABA Concentration in Posterior Cingulate Cortex Predicts Putamen Response during Resting State fMRI

**DOI:** 10.1371/journal.pone.0106609

**Published:** 2014-09-03

**Authors:** Jorge Arrubla, Desmond H. Y. Tse, Christin Amkreutz, Irene Neuner, N. Jon Shah

**Affiliations:** 1 Institute of Neuroscience and Medicine 4, INM 4, Forschungszentrum Jülich, Jülich, Germany; 2 Department of Psychiatry, Psychotherapy and Psychosomatics, RWTH Aachen University, Aachen, Germany; 3 JARA – BRAIN – Translational Medicine, RWTH Aachen University, Aachen, Germany; 4 Department of Neurology, RWTH Aachen University, Aachen, Germany; Hangzhou Normal University, China

## Abstract

The role of neurotransmitters in the activity of resting state networks has been gaining attention and has become a field of research with magnetic resonance spectroscopy (MRS) being one of the key techniques. MRS permits the measurement of γ-aminobutyric acid (GABA) and glutamate levels, the central biochemical constituents of the excitation-inhibition balance in vivo. The inhibitory effects of GABA in the brain have been largely investigated in relation to the activity of resting state networks in functional magnetic resonance imaging (fMRI). In this study GABA concentration in the posterior cingulate cortex (PCC) was measured using single voxel spectra acquired with standard point resolved spectroscopy (PRESS) from 20 healthy male volunteers at 3 T. Resting state fMRI was consecutively measured and the values of GABA/Creatine+Phosphocreatine ratio (GABA ratio) were included in a general linear model matrix as a step of dual regression analysis in order to identify voxels whose neuroimaging metrics during rest were related to individual levels of the GABA ratio. Our data show that the connection strength of putamen to the default-mode network during resting state has a negative linear relationship with the GABA ratio measured in the PCC. These findings highlight the role of PCC and GABA in segregation of the motor input, which is an inherent condition that characterises resting state.

## Introduction

Resting state has become an emerging field of research the understanding of which has brought new insights into brain function. The concept of resting state arose from positron emission tomography (PET) and functional magnetic resonance imaging (fMRI) studies in which the focus moved from stimuli-related brain responses to the spontaneous fluctuations of activity when the brain is not engaged in any particular task [1]–[3]. It was thereby discovered that there exists a high correlation and temporal synchrony of the fMRI blood oxygen level-dependence (BOLD) series among relatively distant brain regions [3]. The analysis of resting state data is possible through independent component analysis (ICA) [4], where the low-frequency patterns of the resting state networks (RSN) are characterised and identified. The default-mode network (DMN) has gained particular interest due to its relationship with neurological and psychiatric conditions [5]–[13] as well as with normal aging [14].

The canonical DMN comprises precuneus, anterior cingulate cortex (ACC), posterior cingulate cortex (PCC), medial prefrontal cortex (MPfC) and lateral parietal inferior gyri (LPIG) [2], [5], [15]. The DMN is thought to characterise basal neural activity [16], [17] and has been linked to self-referential thought, introspection and integration of cognitive and emotional processing [18]. The DMN shows strong activity during rest, as well as rapid deactivation during externally directed tasks [19]. The DMN is also believed to represent an introspectively oriented mode of the mind which provides readiness and alertness to changes in the external and internal environment [15].

The posterior components of the DMN, precuneus and PCC, seem to act as an intrinsic mediatory node of this network [20], [21]. Hagmann et al. [21] used diffusion imaging techniques to demonstrate the existence of a highly connected, complex brain network consisting of the posterior components of the DMN, and showed it to be highly activated at rest. Those regions showed a substantial correspondence between structural connectivity and resting-state functional connectivity.

The PCC has been extensively described as an ‘evaluative region’ [22], and includes Brodmann areas 29, 30, 23, and 31. This region is involved in spatial orientation and memory and it is likely that connections between posterior cingulate and parahippocampal cortices contribute to these processes [22]. Although PCC has been widely investigated, there is no consensus regarding its function [8]. The main functional characterisation of the PCC results from studies which investigate its role within the DMN [23]. PCC is implicated in awareness [24] and internally directed thoughts [13], which is supported by increased PCC activity during internally directed thoughts or during retrieval of autobiographical memories. Importantly, the PCC is one of the areas exhibiting significantly higher activity at rest, as it has been demonstrated by PET and arterial spin labelling [25]. Connectivity studies also demonstrate that the PCC is one of the regions with the highest local functional connectivity in resting conditions [26].

The role of neurotransmitter concentration in the activity of RSN is still not well understood and is an active field of research in magnetic resonance spectroscopy (MRS) [27]. MRS permits the measurement of γ-aminobutyric acid (GABA) and glutamate levels, the central biochemical constituents of the excitation-inhibition balance in vivo. In this sense, the presence of intra-regional and trans-regional neuro-biochemical modulation has been proposed [27]. The latter means that the concentration of a biochemical constituent, as measured by MRS, may predict activity in either the same region, i.e., intra-regionally, and/or another region, i.e., trans-regionally [27]. There is, for example, evidence which suggests the existence of complex interactions between neurotransmitters and the activity of the DMN [28]. Similarly, glutamate measured in the ACC was found to be related with the resting state activity in the same region [29]. The concentration of neurotransmitters has also been related to disease, such as depressive disorder, where abnormal levels of glutamate and GABA have been reported [30].

GABA is the most important inhibitory neurotransmitter in the brain; therefore, it has been linked to several neurological and psychiatric disorders such as epilepsy, panic disorder and depression [31]. Northoff et al. [32] found that the concentration of GABA in the ACC predicts negative BOLD responses of the same area during resting state. In a similar manner, Donahue et al. [33] reported that GABA concentration in the visual cortex is inversely correlated with BOLD signal variations and with cerebral blood flow, suggesting a link between neurochemical and MR-measured hemodynamic responses. In another study, BOLD magnitude was inversely correlated with GABA concentration in the visual cortex, suggesting that the excitation/inhibition cortical balance controls the functional neuroimaging measures [34]. Kapogiannis et al. [35] concluded that regional GABA and glutamate in the posteromedial cortex predict intrinsic functional connectivity of the DMN.

Based on evidence showing the importance of the PCC in the DMN and its importance during resting state [25], as well as the modulatory functions of GABA, we hypothesise that GABA concentration, explicitly the GABA/Cr+PCr ratio – hereafter referred to as the ‘GABA ratio’ – in the PCC measured by MRS has a direct relationship with the response of some areas in the DMN. Previous evidence shows the existence of linear correlations between neurochemicals and the BOLD contrast. Thus, we hypothesise that using a general linear model will reveal clusters exhibiting a linear relationship between the GABA ratio and the neuroimaging metrics measured during resting state, and therefore an assumption of ‘predictability’ could be made. ‘Dual regression’, an analysis tool proposed by Beckmann and co-workers [36], will be used in order to answer these questions. ‘Dual regression’ is a method which permits the identification of between-subject differences in resting functional connectivity [37] based on between-subject similarities using a dual regression approach within the framework of multi-subject-ICA analysis [38].

## Materials and Methods

### Subjects and data acquisition

Data were recorded from 20 healthy male volunteers (mean age = 25.4, SD = 3.7) in a 3 T Siemens Magnetom Trio scanner. Written, informed consent was obtained from all subjects and the study was approved by the Ethics Committee of the Medicine Faculty of the Rheinisch-Westfälischen Technischen Hochschule Aachen (RWTH Aachen University). The study was conducted in accordance with the Declaration of Helsinki. Subjects underwent medical interview and examination in order to exclude psychiatric and neurological conditions. Drug abuse, smoking status and medication intake were assessed using the DIA-X questionnaire (Diagnostisches Expertensystem für Psychische Störungen) [39]. All subjects were right-handed according to the Edinburgh handedness scale [40]. During the scanner procedure the subjects were requested to close their eyes and relax.

Functional images were acquired using a T2*-weighted EPI sequence (TR = 2.2 s, TE = 30 ms, field-of-view = 200 mm, slice thickness = 3 mm and number of slices = 36). The functional time series consisted of 165 volumes. Anatomical images were acquired for every subject by means of a Magnetization-Prepared, Rapid Acquisition Gradient-Echo (MP-RAGE) sequence (TR = 2250 ms, TE = 3.03 ms, field-of-view = 256 × 256 × 176 mm^3^, matrix size = 256 × 256, flip angle = 9°, 176 sagittal slices with 1 mm slice thickness and GRAPPA factor of 2 with 70 autocalibration signal lines).

**Figure 1:**
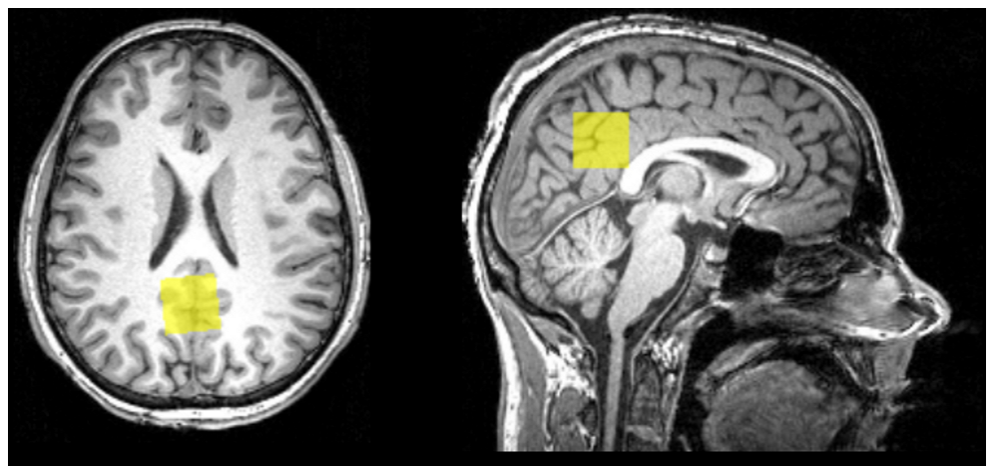
Depiction of voxel positioning for MRS on a background of a T1 individual structural image.

To reliably resolve GABA resonance peaks at 1.9 ppm and 2.3 ppm, single voxel spectra were consecutively measured by standard point resolved spectroscopy (PRESS) with a set of optimised echo times reported by Napolitano et al. [41] (TE1 = 14 ms, TE = 105 ms, TR = 2.5 s, NA = 128, 25 mm × 25 mm × 25 mm voxel size, RF pulse centred at 2.4 ppm, 16 step phase cycling). The duration of the measurement was 5 minutes and 30 seconds. One extra complete phase cycle was measured without the water suppression RF pulse to record a water peak reference for eddy current correction and absolute metabolite concentration calibration. Before the spectroscopy measurements, the static magnetic field was homogenised by running FASTESTMAP [42] iteratively to ensure that the full–width at half maximum (FWHM) of the reference water peak was below 0.05 ppm. The spectroscopy voxel was placed at the PCC by a trained operator (JA). See Figure 1.

### MRS data analysis

The spectra were analysed with LCModel version 6.3-0I [43] using a GAMMA simulated basis set [44]. The simulation was 2D in the two directions where the slice selections were accomplished by 180-degree pulses [45]. The numerical waveforms of the 180-degree pulses were obtained directly from the scanner. The GABA ratio (GABA/Cr+PCr) was extracted and used as covariant in the fMRI resting state data analysis.

### fMRI resting state data analysis

Analysis of functional data was carried out using Probabilistic Independent Component Analysis [38] as implemented in MELODIC (Multivariate Exploratory Linear Decomposition into Independent Components) Version 3.10, part of FSL (FMRIB's Software Library, www.fmrib.ox.ac.uk/fsl). Individual pre-processing consisted of motion correction using MCFLIRT [46], brain extraction using BET [47], spatial smoothing using a Gaussian kernel of FWHM of 5 mm, and high-pass temporal filtering of 100 s. Functional MRI volumes were registered to the structural scan of each individual and standard space (MNI152) images using FLIRT [46], [48]. Temporal concatenation ICA was performed across all functional datasets from each subject using automatic dimensionality estimation [38]. The DMN was identified by visual inspection and comparison to previously published data [2], [49] Finally, the dual regression algorithm [36] was applied to the ICs in order to identify the individual contribution of every subject to the RSNs using the GABA ratio in the PCC as a covariant in the second stage of dual regression analysis within the framework of the general linear model. Here, the subject-specific GABA ratio was tested for linear relationship with the subject-specific z–values of the IC representing the DMN. The different component maps were collected across subjects into single 4D files and tested voxel-wise for statistically significant correlation using nonparametric permutation testing (10000 permutations) [50] and ‘threshold-free cluster enhancement’ for improved sensitivity [51]. This resulted in spatial maps characterising the voxels with signal intensities that had a linear relationship (slope) with GABA ratio. The maps were thresholded and controlled for family-wise error rate at p<0.05 [52].

Additionally, a Pearson product-moment correlation was used to test whether the GABA ratio measured in the PCC had any relationship with the absolute and relative motion observed during the acquisition of the fMRI data.

## Results

All the subjects reported full compliance with the instructions; no self-reports of having fallen asleep were given.

**Figure 2:**
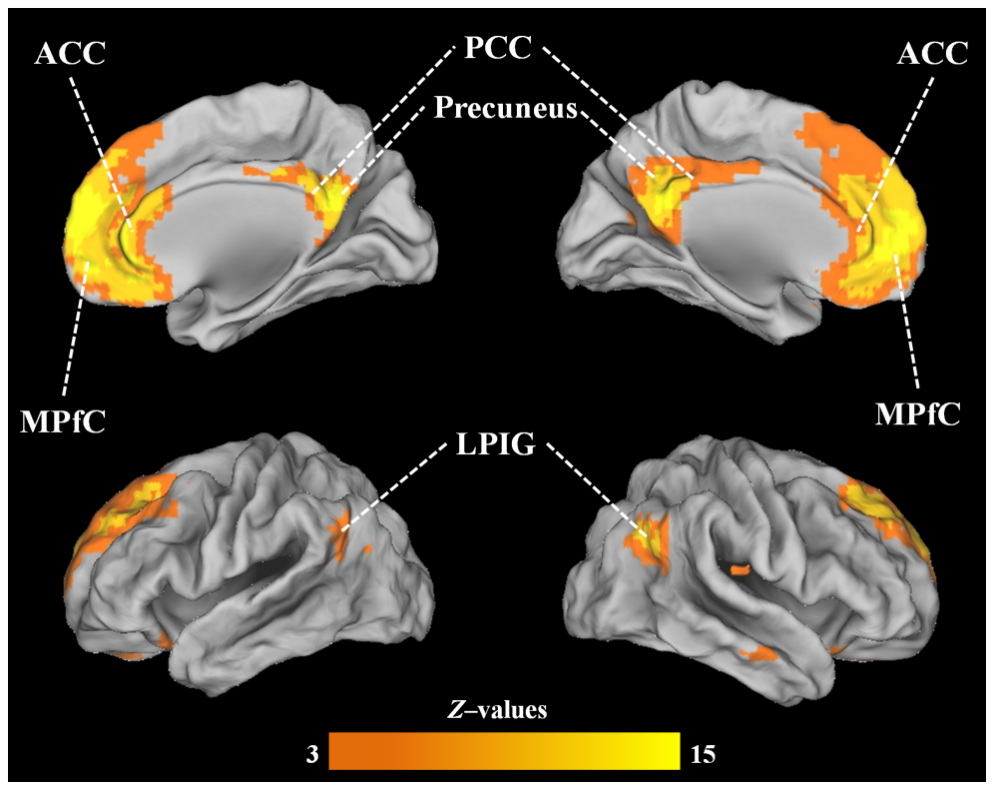
DMN identified in the group analysis of the 20 subjects; the DMN was picked by visual inspection, comprising medial prefrontal cortex (MPfC), anterior and posterior cingulate cortices (ACC and PCC), precuneus and lateral parietal inferior gyri (LPIG).

Twenty-three components were found after decomposition of the data by means of ICA. ‘Meaningful’ RSNs (i.e. representing neuronal signal as opposed to physiological and non-physiological noise such as vascular, respiratory and motion artefacts) were identified by matching them visually against a previously published set of data encompassing 20 ‘canonical’ RSNs (http://fsl.fmrib.ox.ac.uk/analysis/brainmap+rsns) [49]. In accordance with the study by Smith et al. [49] the following 9 RSNs were identified in our data: right and left frontoparietal networks, medial visual network, occipital pole visual network, lateral visual network, DMN, auditory network, executive control network and sensorimotor network. The DMN was picked by visual inspection, comprising medial prefrontal cortex, anterior and posterior cingulate cortices, precuneus and lateral parietal inferior gyri [2], [49]. See Figure 2.

The GABA ratio was successfully measured in the PCC using single voxel spectra (mean = 0.177, SD = 0.024).

**Figure 3:**
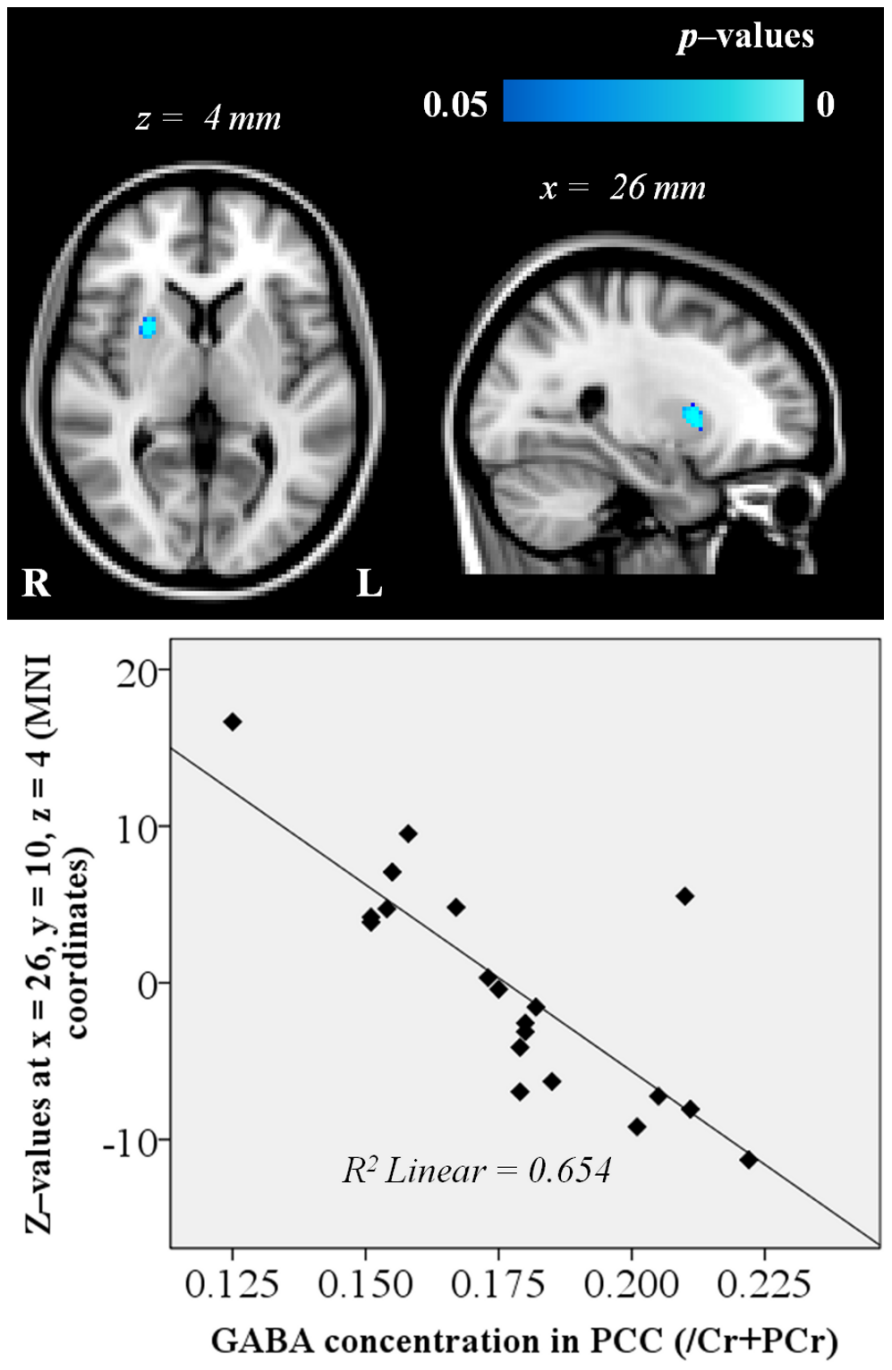
Voxel-wise statistical maps with the inclusion of the GABA ratio measured in the PCC and generated by the dual regression analysis of the DMN. Significant voxels thresholded at p<0.05 (corrected). The point of lowest p value (p = 0.0002) was located in MNI coordinates x = 26, y = 10, z = 4. The background image is the MNI152 T1 (2 mm) template.

The voxel-wise statistical maps, generated by the permutation test of dual regression, exhibited a cluster in the right putamen with significant values (p<0.05, corrected) where the connection strength within or to the DMN had a negative linear relationship with GABA ratio measured in the PCC. See Table 1. The point of lowest p value (p = 0.0002) was located in the right putamen (MNI coordinates x = 26, y = 10, z = 4) according to the Harvard-Oxford Subcortical Structural Atlas (Figure 3).

**Table 1:** MNI coordinates (2 mm template) of clusters with minimal P values of linear relationship between GABA ratio and z–values in the DMN.

Number of significant voxels in the cluster	Regions of minimum p value according to the Harvard-Oxford Cortical Structural Atlas	Min. corrected *P* value	MNI coordinates		
			*x*	*y*	*z*
65	96% Right Putamen, 4% Right Cerebral White Matter	0.0002	26	10	4

The Pearson product-moment correlation between the GABA ratio and the absolute motion during the acquisition of the fMRI data was *r*(18) = −0.321, *p* = 0.168. In the case of the GABA ratio and the relative motion the correlation coefficient was *r*(18) = −0.411, *p* = 0.072.

**Figure 4:**
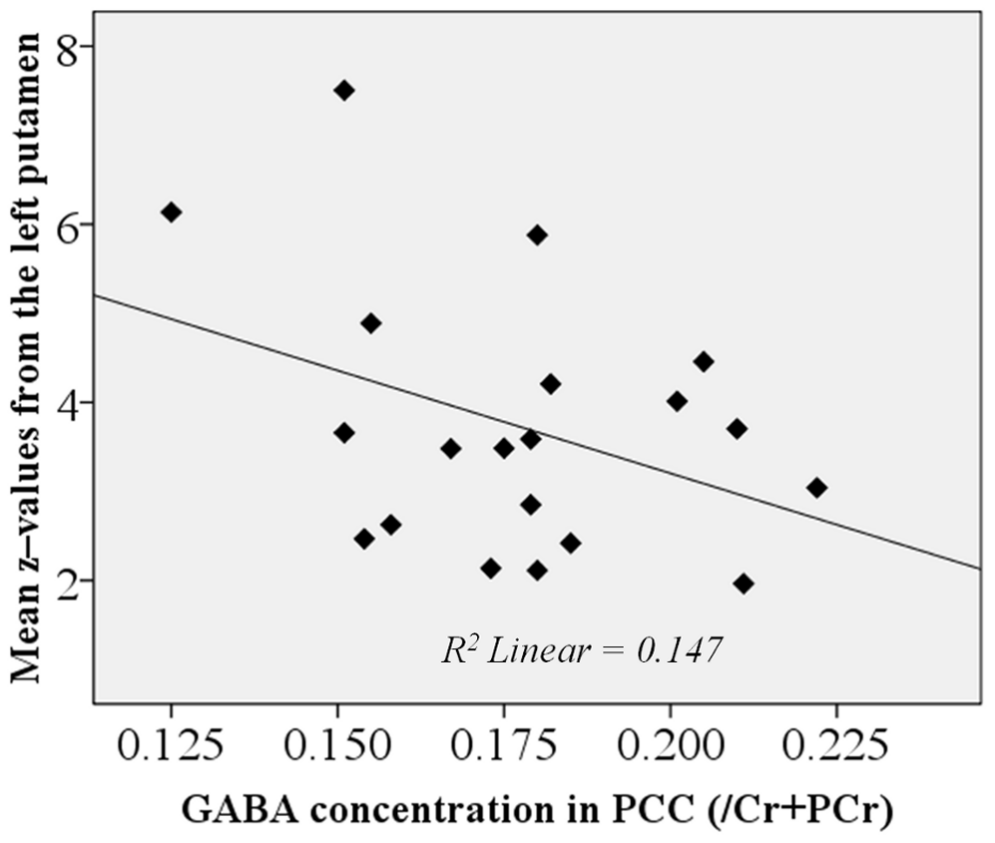
Mean z–values extracted from the left putamen in relation to GABA ratio in the PCC.

Additionally, a region-of-interest analysis was performed in order to test whether a similar relationship between GABA ratio and signal intensity in the left putamen could be found. The mean z–values of the individual DMN maps were extracted from the left putamen according to the Harvard-Oxford Subcortical Structural Atlas and tested for correlation with the GABA ratio using the Pearson product-moment correlation. The correlation coefficient was *r*(18) = −0.383, *p* = 0.095 (Figure 4).

## Discussion

Given the prior evidence of the modulatory role of GABA in the excitation/inhibition balance we investigated its role in the PCC, an important hub of the DMN. We conducted a study in healthy male volunteers in which the GABA ratio was measured in the PCC using MRS at 3 T. The measured values were included in the analysis of fMRI resting state data in order to identify the relationship between the GABA ratio values and the response of the DMN. Our results show that the activity of the right putamen in the DMN has a negative linear relationship with the GABA ratio measured in the PCC.

The results presented here confirm previous observations in which GABA concentration had an inverse relationship with the magnitude of BOLD signal [32], [33], and suggest that the putamen is a structure whose activity during resting state is intrinsically modulated by the concentration of GABA. Previous investigations already showed how regional BOLD signal appears to be governed by local GABA concentration either in the same or other regions [27], [32]–[34].

The negative relationship found between GABA ratio and the right putamen response seems to be also extended to the left putamen; although in our data it did not achieve statistical significance probably due to the number of participants and the strictness of the statistical model used for the voxel-wise analysis.

Noteworthy is the fact that the putamen is not part of the DMN, although it has been described as being part of a basal ganglia RSN, which corresponds to the motor control circuit [53], [54]. The interactions among RSNs have been extensively described, particularly for the case of the DMN, which is the network holding hubs with the highest global functional connectivity [55]. A recent fMRI study suggests the presence of complex modulatory interactions among the DMN and the other networks in resting state. Such communications among networks seem to be modulated by critical brain structures such as the basal ganglia and the thalamus [56]. Moreover, there is evidence of negative interactions between the basal ganglia and the activity of the DMN [56]. In this regard, Tomasi et al. [53] described the existing segregation of the DMN from the other networks, which appears to be necessary for its deactivation during task performance. The results presented here suggest that GABA is a critical neurotransmitter for the interactions among RSNs, particularly for the case of the DMN and the basal ganglia RSN, where putamen is a relevant structure.

The importance of the putamen in the activity of the DMN has also been previously described. In a PET study Tomasi et al. [57] demonstrated that the availability of dopamine and dopamine transporters in the putamen had a negative linear correlation with deactivation in areas belonging to the DMN. Moreover, the putamen has been included as one of the regions with higher local functional connectivity density at rest [26], and there is evidence of segregation towards this structure to slow the access to cortical sources [55].

Functions of the putamen are majorly categorised as ‘motor functions’. It has a well-known role in motor preparation, execution and control [58]. In an fMRI study it was established that putamen is a target area for proactive motor inhibition driven by the MPfC and the inferior parietal cortex [59]. Among the functions of putamen, learning and memory processes have also been described [60], particularly in studies where lesions of the putamen impair visual discrimination and learning in non-human primates [61]. Further evidence on the functions of the putamen is provided by the pathophysiology of Parkinson's and Huntington's disease, both exhibiting a variety of cognitive deficits as well as cell loss in the putamen. Nigrostriatal projection loss with dopamine deficiency in the putamen is the feature that characterises Parkinson's disease [62], while atrophy patterns in the putamen have been described at different stages of Huntington's disease [63].

Modulatory functions of neurotransmitters in relation to the putamen have also been described. In an animal study, Packard [64] demonstrated that rats which received an infusion of glutamate in the caudate-putamen exhibited increased place learning which influenced behaviour. This evidence suggests that the learning functions modulated by the putamen might depend on the concentration and/or balance of neurotransmitters. Our results extend the mutual modulatory role over the putamen also to GABA, although just in the sense of segregation of the motor input that occurs during resting state and that is driven by the activity of the DMN. Our results add to the existing evidence that GABA is an important neurotransmitter with diverse functions during resting state [32], [33]. The results presented here highlight the role of GABA in the segregation of the motor engagement, which is an inherent condition that characterises resting state.

The inhibitory functions of GABA have also been well-described [65]. GABAergic systems mediate most fast synaptic inhibition in the mammalian brain, controlling activity at both the network and the cellular levels. There is evidence of the role of GABA in motor inhibition [66], and moreover, our results highlight its inhibitory functions during resting state. Based on the best evidence our study included only male volunteers. The effect of female gonadal steroids on GABAergic systems is well-known and has been described as a modulator factor [67]. In a MRS study, Epperson et al. [68] found a reduction in cortical GABA levels during the follicular phase of the human menstrual cycle, and therefore, this explorative study included only male volunteers.

Inhibitory functions direct multiple processes and networks in the central nervous system. In an fMRI study, Jaffard et al. [59] identified a network involved in motor inhibition which included the superior parietal lobule, PCC, precuneus, parahippocampal gyrus and thalamus. Some of these areas also belong to the DMN; hence they conclude that the resting state activity, which is not directly related to identifiable sensory or motor events, controls the balance between excitatory and inhibitory activations determining responsiveness to possible incoming events [59]. The PCC has been identified as a key structure in inhibition tasks with a possible role in alertness. Furthermore, activation of the putamen was identified when motor inhibition was expected to be ‘on’. Additionally, Hu and Li [69] confirmed that the PCC and putamen were both identified as areas involved in preparatory motor execution during rest. Moreover, dense anatomical connections between the PCC and the striatum have been described [8], supporting the role of PCC in directing the segregation of the motor input.

The conclusion in the study by Jaffard et al. [59], according to which the activity at rest may be partly due to an active and sustained process consisting of locked movement initiation mechanism, is in line with our results and, moreover, we hypothesise that this mechanism is linked to the concentration of GABA. The fact that the concentration of GABA in the PCC, the ‘core’ of resting state, predicts the BOLD response of the putamen supports this hypothesis.

Even though our results appear plausible from a physiological point of view, some limitations must be mentioned. Unfortunately, the measures that each of the modalities provides are not necessarily complementary [27]. Furthermore, the concentrations of transmitters measured by MRS reflect also pools that are not used for neurotransmission, making it more difficult to understand the exact mechanisms of the neuro-biochemical relationships. Hence, the approach described here does not permit to answer questions of causality.

GABA has a concentration of about 1 mM in human brain. This is about an order of magnitude lower than that of some other metabolites and is about 40,000 times lower than the concentration of water [70]. At 3 T, resonance peaks of GABA at 1.9 ppm, 2.3 ppm and 3.0 ppm overlap with the large resonance peaks from n-acetyl aspartate (NAA), total glutamate and glutamine (Glx), and creatine (Cr), respectively. These overlaps make the detection of GABA error-prone. Currently, the most commonly applied MRS method to separate the GABA signal from the rest of the spectrum is Mescher-Garwood point resolved spectroscopy (MEGA-PRESS), in which the J-coupling between GABA-H3 at 1.9 ppm and GABA-H4 at 3.0 ppm is exploited by an on/off frequency selective RF pulse applied at 1.9 ppm [71]. Due to the lack of J-coupling at 1.9 ppm for Cr, its signal at 3.0 ppm is removed by taking the difference from the two sets of measurements, leaving only the GABA-H4 peaks at 3.0 ppm. This method relies on the subtraction of two sets of spectra to remove any strong overlapping peaks; hence, it is vulnerable to any instability caused by the scanner, e.g., magnetic field drift, or by the subject, e.g., movement.

Another popular method to detect GABA is two-dimensional MRS [72] in which a series of spectra that differ by a single parameter, such as a delay duration or the timing of a refocusing pulse, are acquired. The second spectral dimension contains the coupling information which, in turn, allows overlapping multiplets to be resolved. However, these experiments usually require longer acquisition time due to the increased number of measurements. Recently, Napolitano et al. [41] demonstrated that using a standard PRESS sequence with a set of optimised echo time parameters, they could reliably detect GABA in the ACC and the precuneus region in a shorter measurement time and in a smaller voxel size than previous studies with MEGA-PRESS. Due to the time constrain of the multi-modality investigation in this study, the standard PRESS sequence with optimised echo time parameters was chosen to detect GABA.

The use of GABA ratio in the present study instead of absolute concentration is in agreement with previous publications [33], [35], in which metabolite levels are commonly reported as their ratio to creatine. Creatine has been shown to be a stable metabolite in healthy individuals [73] and thus is commonly used as an internal reference in brain spectroscopy. Furthermore, in a study by Bogner et al. [74] GABA ratio exhibited the best reproducibility.

From a technical point of view, we must remark that the separation and quantification of metabolites are often ambiguous and difficult due to several factors. First, since 80% of brain tissue is endogenous water, the water resonance peaks is several orders of magnitude higher than that of the metabolites, which can potentially distorts nearby metabolite resonance signals. Second, large subcutaneous lipids signals can potentially ruin metabolite signals. Third, the spectral resolution as well as the signal-to-noise ratio are often reduced by the anatomical induced magnetic field spatial inhomogeneity. Despite these difficulties were overcome in this study, the process of separating metabolite signals is still challenging because of the large number of overlapping metabolite resonance peaks confined in a narrow chemical shift range of ∼4 ppm.

## Conclusions

The results presented here show that the connection strength of putamen to the DMN during resting state has a negative linear relationship with the GABA ratio measured in the PCC. These findings highlight the role of PCC and γ-aminobutyric acid in the segregation of the motor input that occurs during resting state. Our data support the notion that the activity of the DMN implies deactivation of the motor control circuit, which corresponds to the basal ganglia RSN, where putamen is a relevant structure. This study confirms once more the possibility and utility of measuring local concentration of transmitters using MRS.
